# Exploring the molecular mechanisms and therapeutic potential of SMAD4 in colorectal cancer

**DOI:** 10.1080/15384047.2024.2392341

**Published:** 2024-08-20

**Authors:** Hui Shan, Guangyu Tian, Yeqing Zhang, Zhiyuan Qiu

**Affiliations:** a Department of Oncology, the Affiliated People’s Hospital of Jiangsu University, Zhenjiang, Jiangsu, China; b Department of Oncology, Jiangdu People’s Hospital Affiliated to Yangzhou University, Yangzhou, Jiangsu, China; c Department of Vascular Surgery, the Second Affiliated Hospital of Soochow University, Suzhou, Jiangsu, China

**Keywords:** SMAD4 protein, CRC, Tgf-β pathway

## Abstract

Colorectal Cancer (CRC) is the third most common cancer worldwide, and the occurrence and development of CRC are influenced by the molecular biology characteristics of CRC, especially alterations in key signaling pathways. The transforming growth factor-β (TGF-β) plays a crucial role in cellular growth, differentiation, migration, and apoptosis, with SMAD4 protein serving as a key transcription factor in the TGF-β signaling pathway, thus playing a significant role in the onset and progression of CRC. CRC is one of the malignancies with a high mortality rate worldwide. Despite significant research progress in recent years, especially regarding the role of SMAD4, its dual role in the early and late stages of tumor progression has promoted further discussion on its complexity as a therapeutic target, highlighting the urgent need for a deeper analysis of its role in CRC. This review aims to explore the function of SMAD4 protein in CRC and its potential as a therapeutic target.

## 1. Introduction

Colorectal Cancer (CRC) is the third most common cancer worldwide and the second leading cause of cancer-related deaths^1^. The occurrence and development of CRC are influenced by a combination of genetic and environmental factors. The prognosis of CRC is also influenced by various factors, including tumor stage, molecular characteristics, and treatment response. Among these factors, the molecular biology characteristics of CRC are particularly important, especially alterations in key signaling pathways. The transforming growth factor-β (TGF-β) signaling pathway plays a complex role in normal intestinal physiology and tumorigenesis, and its imbalance is closely associated with poor prognosis in CRC. The SMAD4 protein, as a core component of the TGF-β signaling pathway, plays a critical role in regulating cell proliferation, differentiation, and apoptosis. Although current treatment methods have improved the survival rate of CRC patients to some extent, finding more effective treatments remains a significant clinical challenge, especially for patients with adverse molecular markers, such as SMAD4 mutations.^2^

Given the importance of SMAD4 protein in CRC, this review will explore the role of SMAD4 protein in the development of CRC and its potential molecular mechanisms. We will analyze how SMAD4 protein regulates tumor growth, apoptosis, angiogenesis, and invasiveness by affecting the TGF-β signaling pathway. In addition, we will discuss the application of SMAD4 protein as a biomarker in the diagnosis and prognosis of CRC, as well as the potential value of subsequent therapeutic strategies targeting SMAD4 protein in the treatment of CRC. Through a comprehensive analysis of the role of SMAD4 protein in CRC, this review aims to provide new perspectives and directions for the diagnosis, treatment, and future research of CRC.

## 2. SMAD4 gene and its protein product

### 2.1. Structure and function of SMAD4 protein

The SMAD4 protein acts as a co-mediator involved in regulating processes such as cell proliferation, differentiation, and apoptosis. The SMAD4 gene is a critical member of the TGF-β signaling pathway, which plays an important role in maintaining the normal physiological functions of cells. Research has shown that alterations in SMAD4 are closely associated with the development of various gastrointestinal tumors, including gastric cancer, CRC, and pancreatic cancer.^3–5^

In CRC, SMAD4 plays a key role in the TGF-β signaling pathway, which regulates cell proliferation, differentiation, and apoptosis. Alterations in SMAD4, including downregulation or loss of protein expression, are associated with increased tumor and metastatic ability, and these changes are often closely associated with poor prognosis in CRC patients. However, the effect of SMAD4 is not a direct cause of increased tumor invasiveness but rather by affecting multiple aspects of the TGF-β signaling pathway and regulating the expression of downstream target genes, thereby comprehensively influencing tumor behavior. Thus, alterations in SMAD4 are an important factor affecting the CRC in progression, influencing tumor characteristics by participating in the regulation of multiple biological processes.^6^ Typically, SMAD4 interacts with other SMAD proteins to facilitate the transduction of TGF-β signals from the cell surface to the nucleus, thereby affecting the expression of target genes. Studies have shown that SMAD2, another member of the SMAD family, has a high correlation coefficient, suggesting a synergistic effect in tumor suppression.^7,8^ However, most CRC patients have mutations or decreased expression of the SMAD4 gene occur, which leads to impairment of the TGF-β signaling pathway, thereby promoting tumor proliferation and increased invasiveness.

In addition to the classical TGF-β/SMAD4 pathway, SMAD4 also participates in other signaling pathways, such as interacting with the bone morphogenetic protein (BMP) signaling pathway, to co-regulate biological processes such as cell proliferation and differentiation.^9,10^ Additionally, SMAD4 can interact with the Wnt/β-catenin signaling pathway to influence cell fate determination and tissue regeneration.^11^ Furthermore, in certain contexts, SMAD4 can also interact with the Notch signaling pathway and participate in cell differentiation and development processes.^12^

In summary, as a highly significant transcription factor, SMAD4’s function is not limited to the TGF-β signaling pathway alone. Through interactions with multiple signaling pathways, it is extensively involved in various aspects of cellular life activities, highlighting the breadth of its functions.

### 2.2. The role of SMAD4 protein in the tgf-β signaling pathway

The TGF-β signaling pathway plays a complex role in the development of CRC, acting as a double-edged sword that can both inhibit tumor formation and promote tumor progression.^13,14^ SMAD4 protein plays a pivotal role in this pathway, where its loss of function not only affects cell cycle regulation and apoptosis but can also lead to cellular evasion of growth inhibitory signals, thereby promoting tumor development. Moreover, the status of SMAD4 influences the normal function of the TGF-β signaling pathway, whose aberrant activation contributes to the shaping of the tumor microenvironment, promoting tumor cell invasion, migration, and neovascularization, ultimately leading to the deterioration and poor prognosis of CRC.^15^

As mentioned above, the SMAD4 gene is a key member of the TGF-β signaling pathway. SMAD4 forms a complex with receptor-regulated Smads (R-Smads) and together they enter the nucleus to regulate the transcription of target genes. In the primary process, the ligand TGF-β binds to serine/threonine kinase receptors (type I and II) on the cell surface, inducing receptor phosphorylation. These activated receptors then phosphorylate selected Smads at the C-terminus, whereupon these receptor-activated R-Smads form a complex with SMAD4. These activated Smad complexes translocate to the nucleus, where they regulate the transcription of target genes through physical interactions and functional cooperation with DNA-binding transcription factors.^14^

In addition to the classical SMAD-dependent pathway described above, TGF-β also involves SMAD-independent pathways that can signal through other factors such as TRAF4, TRAF6, TAK1, etc., affecting processes such as apoptosis, migration, and proliferation.^16–19^ Although SMAD4 is primarily involved in the classical signaling pathway, it can also indirectly affect non-classical pathways through interactions with other signaling pathways.^20^

In summary, SMAD4 serves as a critical center for signaling and gene expression regulation within the TGF-β signaling pathway. Its function and role are critical for the normal functioning of this pathway.

### 2.3. Research on SMAD4 gene mutations and dysregulation in other cancers

The mutation and dysregulation of the SMAD4 gene are widely present in various cancers, sparking research interest in whether this gene also plays a crucial role in CRC.

Studies have shown that alterations in SMAD4 are closely associated with the occurrence, progression, and prognosis of multiple cancers. For example, in pancreatic cancer, the loss of SMAD4 is a common event, and its absence is associated with increased aggressiveness and poor prognosis of the disease. Approximately 55% of pancreatic cancer patients have loss or downregulation of SMAD4, which is considered a key factor in the malignant progression of pancreatic cancer.^21^ Research involving sequencing for SMAD4 gene mutations and homozygous deletions has revealed genetic abnormalities in pancreatic cancer, with SMAD4 mutations being prevalent in a large number of cases.^22^ In addition, studies have shown that the inactivation of the SMAD4 gene is associated with poorer prognosis in patients who have undergone surgical resection of pancreatic cancer.^23,24^

Mutations in SMAD4, as well as the loss or reduction of its expression, have been found to be closely associated with the progression and survival of patients with non-small cell lung cancer (NSCLC).^8^ Furthermore, in hepatocellular carcinoma (HCC), loss of SMAD4 has been implicated as a mechanism by which HCC cells gain invasive and metastatic capabilities. Mutations or dysregulation of the SMAD4 gene in HCC patients are associated with a poorer survival rate. In addition, research has shown that SMAD4 point mutations occur in a very small number of cases of kidney cancer, esophageal cancer, and cholangiocarcinoma.^25–27^ Although such point mutations are rare, other drivers, such as alternative splicing and protein loss, are common in cancer, thus providing some explanation for these types of mutations.

All these findings reveal the important role of the SMAD4 gene in various cancers and suggest that its potential similar role in the development of CRC should be explored. Additionally, studies have indicated that it plays a significant role in CRC.^2,28^ By further comparing the functions and related mechanisms of SMAD4 in various cancers, new targets for future treatment strategies may be identified.

## 3. The role of SMAD4 protein in CRC

### 3.1. The Relationship Between SMAD4 Gene Mutation, dysregulation, and CRC

The relationship between SMAD4 gene mutation, dysregulation, and CRC has been extensively studied. In CRC, mutations in the SMAD4 gene are often closely associated with the aggressiveness of the disease, increased ability to metastasize, tumor location, and poor prognosis of CRC patients.^2,29^ Numerous studies have shown that patients with CRC lacking the SMAD4 gene tend to have a worse prognosis compared to those without this deletion, exhibiting shorter recurrence-free survival (RFS). This suggests that the absence of the SMAD4 gene represents a unique subtype within CRC. Research on CRC indicates that the molecular characteristics of the cancer are significantly related to location of the tumor, which may indirectly influence the relationship between SMAD4 deletion and the development of CRC. The SMAD4 gene acts as a tumor suppressor gene, inhibiting the proliferation, invasion, and metastasis of tumor cells. In contrast, the loss or dysfunction of the SMAD4 gene promotes tumor initiation and progression through various mechanisms.^2,30^

Firstly, from the perspective of mutations, the mutation of the SMAD4 gene represents a significant genetic variation in CRC. Systematic reviews have shown that mutations in the SMAD4 gene are relatively common in CRC. These mutations often affect the normal function of the SMAD4 protein, especially its role in TGF-β signaling. Such mutations often affect structural domains of the SMAD4 protein, such as the DNA-binding region, thereby preventing its effective binding with the TGF-β receptor, interrupting the normal TGF-β signaling pathway, and disrupting the tumor suppression mechanism, which promotes the formation and progression of tumors.^2^

Secondly, the dysregulation of SMAD4 gene expression is also closely related to the occurrence and development of CRC. Studies have shown that, the expression of SMAD4 is significantly reduced or completely absent in CRC.^31,32^ This dysregulation not only weakens the function of SMAD4 in inhibiting tumor cell proliferation, invasion, and metastasis but also may lead to the deterioration of the tumor microenvironment, thereby promoting tumor metastasis and the emergence of drug resistance. The loss or functional impairment of the SMAD4 gene promotes tumor initiation and progression through multiple mechanisms. First, as described above, SMAD4 is a key regulator in the TGF-β signaling pathway. When it is lost or inactivated, TGF-β signal transduction is disrupted, leading to abnormalities in cell cycle regulation and apoptosis.^14^ Second, the loss of SMAD4 alters cell-to-cell communication and signal transduction, particularly its interactions with other signaling pathways such as Wnt, MAPK, and PI3K/AKT, thereby increasing the invasiveness and migratory abilities of tumor cells.^15^ Additionally, the loss of SMAD4 is associated with changes in the tumor microenvironment, including promoting the accumulation of tumor-associated macrophages and the formation of an immune-suppressive microenvironment. These mechanisms collectively contribute to tumor initiation, progression, and poor prognosis.^33,34^

Therefore, whether it is a functional loss caused by gene mutations or abnormal expression, SMAD4 plays a crucial pathophysiological role in the onset and development of CRC. Therefore, understanding the specific mechanisms of the SMAD4 gene in CRC is of great importance for improving the treatment outcomes and quality of life of CRC patients.

### 3.2. The impact of SMAD4 on CRC cell proliferation, migration, and invasion capability

The SMAD4 gene plays a central regulatory role in the pathogenesis of CRC, significantly impacting the proliferation, migration, and invasion capabilities of CRC. As a core component of the TGF-β signaling pathway, the normal function of SMAD4 helps maintain the balance of cell growth and prevents uncontrolled cell proliferation.

In terms of cell proliferation, SMAD4 mediates tumor-suppressive signals under normal circumstances, thereby inhibiting the excessive proliferation of tumor cells. However, when SMAD4 is mutated or its expression is downregulated, its negative regulatory effect on the cell cycle will be weakened. This causes CRC cells to lose their normal growth control mechanisms, further promoting the rapid tumor proliferation.^2,35^

Mutations and dysregulation of SMAD4 also enhance the migration and invasion capabilities of CRC cells. Studies have shown SMAD4 can influence cell adhesion and morphological changes by regulating the expression of genes related to the epithelial-mesenchymal transition (EMT) process, such as E-cadherin and N-cadherin, thereby affecting the metastasis and spread of tumor cells from the primary site to distant tissues.^35–40^ SMAD4 plays a central role not only in regulating the migration and invasion of CRC cells but also in regulating the expression of many key molecules and enzymes closely associated with these processes.^41^ In particular, SMAD4 significantly affects the expression of members of the matrix metalloproteinases (MMPs) family, which can degrade various components of the extracellular matrix (ECM). By degrading ECM, these enzymes pave the way for tumor migration and invasion, further promoting the spread of the tumor.^42^

Loss of SMAD4 function can lead to enhanced c-MYC activity. c-MYC, in turn, upregulates the expression of NLE1, a ribosome biogenesis factor. The upregulation of NLE1 enhances the protein biosynthesis capacity of colorectal cancer cells, which is crucial for tumor cell growth and metastasis.^31^ Therefore, the loss of SMAD4 function promotes CRC growth and metastasis. Furthermore, studies have shown that in CRC, a mutation of arginine at position 361 of SMAD4 (SMAD4 R361) not only disrupts the binding of SMAD4 to phosphorylated SMAD2/3, thereby hindering normal TGF-β signal transduction and weakening TGF-β’s growth-inhibitory and apoptosis-inducing functions, but also potentially enhances SMAD4 binding to Lef1 protein. This interaction activates the Wnt/β-catenin signaling pathway, thereby driving cancer progression and exacerbation.^29^

In summary, the role of SMAD4 in CRC is not limited to inhibiting tumor cell proliferation but also includes the regulation of tumor cell migration and invasion capabilities. Mutations or dysregulation of SMAD4 not only promote the uncontrolled proliferation of CRC cells but also enhance their ability to migrate and invade, thereby accelerating the disease progression.^35,43^ A deeper understanding of the specific mechanisms by which SMAD4 influences the biological behavior of CRC cells is crucial for developing new therapeutic strategies and delaying disease progression.

### 3.3. The regulatory role of SMAD4 in the CRC microenvironment

The role of SMAD4 in the CRC microenvironment extends beyond its function as a tumor suppressor. Given the critical role of the TGF-β signaling pathway in various physiological and pathological processes, including cell proliferation, differentiation, apoptosis, and immune response regulation, SMAD4 plays an important role in these processes. In particular, the absence or dysfunction of SMAD4 has a significant impact on the regulation of the CRC microenvironment.

Recent studies have indicated that SMAD4 deficiency may potentially increase tumor immunogenicity by altering the expression of immune-modulating factors, including cytokines, chemokines, and immune checkpoint molecules like PD-L1, and by altering the release of tumor cell surface antigens, thus impacting the interactions between tumor cells and the host immune system.^2,40^ Additionally, the inactivation of SMAD4 could lead to an increased state of local immune suppression, further allowing tumor cells to evade surveillance and elimination by the body’s immune system. This could manifest as a reduction in tumor-infiltrating lymphocytes, upregulation of immune checkpoint molecules, and dysregulation of pro-inflammatory cytokine secretion.^35,40^

Studies have shown that SMAD4 exerts its tumor-suppressive effects in CRC by downregulating the expression of the key inflammatory mediator CCL20. CCL20, a chemokine, attracts immune cells such as T cells and dendritic cells into the tumor microenvironment through its receptor CCR6. When SMAD4 is lost in CRC, the expression of CCL20 and CCR6 is significantly increased, leading to a higher influx of immune cells, promoting an inflammatory milieu that facilitates CRC progression.^44^

In the regulation of the CRC microenvironment, SMAD4 also plays a critical role in the activation and maintenance of cancer-associated fibroblasts (CAFs),^15,45,46^ which produce a significant amount of matrix components in the fibrotic microenvironment of CRC and enhance tumor invasiveness and chemotherapy resistance.^47^ The absence of SMAD4 can further promote the fibrotic process around the tumor, form a physical barrier conducive to tumor dissemination and induce angiogenesis to meet the needs of rapidly proliferating tumor cells.^48–50^

**Figure:**
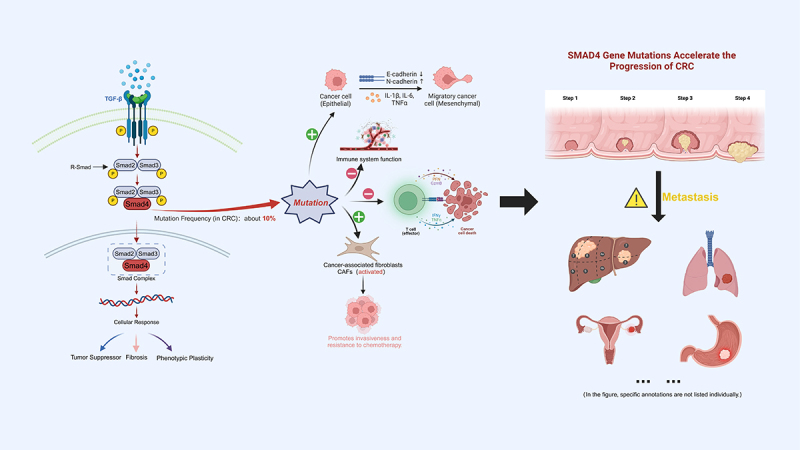

SMAD4 also plays a vital role in regulating the immune microenvironment of CRC, including influencing macrophage polarization of macrophages,^51,52^ and the function of natural killer cells, dendritic cells, and T cells.^53^ Loss of SMAD4 may induce immune cells to adopt a pro-tumorigenic phenotype, thus worsening patient prognosis.^54,55^

Furthermore, SMAD4 indirectly shapes a tumor-promoting microenvironment through the transcriptional regulation of inflammatory factors and the impact on tumor cell metabolism.^56^ For example, the absence of SMAD4 may lead to an abnormal increase in glycolysis and fatty acid metabolism in tumor cells.^40,57,58^ These metabolic changes not only support tumor cell proliferation but may also affect the function of immune cells, further modulating core immune responses.^59^

In summary, the regulatory role of SMAD4 in the CRC microenvironment is complex and multidimensional. It profoundly affects the development and prognosis of CRC through its impact on immune response regulation, tumor-stroma interactions, immune cell function, and inflammatory metabolism regulation. Understanding the role of SMAD4 in regulating the CRC microenvironment is crucial for developing new therapeutic strategies and improving patient outcomes.

## 4. The potential of SMAD4 as a biomarker for CRC

### 4.1. The Application of SMAD4 in Diagnosis, prognostic assessment, and treatment response monitoring

The SMAD4 gene and its protein play an important role in the management of CRC, particularly in the diagnosis, prognostic assessment, and monitoring treatment responses.

In the diagnosis of CRC, the expression and localization of SMAD4 protein can be detected through techniques such as immunohistochemistry (IHC). Research on Juvenile Polyposis Syndrome (JPS) has shown that SMAD4 IHC, as an indicator of SMAD4 gene status, can serve as a preferred screening method for molecular diagnosis,^60^ which is crucial for distinguishing CRC from other types of intestinal diseases. Since mutations or dysregulation of SMAD4 are relatively common in CRC, the detection of SMAD4 status can serve as an important adjunct indicator for pathological diagnosis.

The status of SMAD4 is closely related to the prognosis of CRC patients. Studies have shown that the absence or reduced expression of SMAD4 is often associated with higher tumor stages, increased aggressiveness, and metastatic potential.^2,61–63^ Moreover, patients with reduced expression of SMAD4 tend to have shorter disease-free survival (DFS) and overall survival (OS), making SMAD4 an effective independent prognostic factor for predicting patient survival probabilities and risk of disease progression.^64^

As the understanding of SMAD4‘s mechanisms of action deepens, its potential to guide personalized treatment strategies is increasingly recognized. In CRC patients undergoing chemotherapy or radiotherapy, changes in SMAD4 levels may indicate the degree of tumor response to treatment. For example, monitoring SMAD4 levels in serum or tissue could reflect the response of CRC patients to postoperative adjuvant chemotherapy. Related research indicates that mutations in SMAD4 may serve as a potential biomarker for poor prognosis with cetuximab (an anti-EGFR monoclonal antibody) based therapy.^65^ Some studies have even suggested SMAD4 as a candidate biomarker for combination chemotherapy regimens based on LY294002 (a PI3K inhibitor) and 5-fluorouracil (5-FU), with SMAD4 deficiency possibly being associated with resistance to 5-FU based therapy.^66,67^ Additionally, considering SMAD4‘s role in regulating the TGF-β signaling pathway and its immunosuppressive effect in the microenvironment, targeted therapies addressing SMAD4 dysfunction are being developed, offering new directions for treatment. Therefore, monitoring the status of SMAD4 can help evaluate the efficacy of new therapeutic strategies.

In conclusion, SMAD4 holds significant application value in the diagnosis, prognostic assessment, and treatment response monitoring of CRC. Investigation of the specific roles of SMAD4 in CRC is crucial for improving diagnostic accuracy, predicting disease progression, and optimizing treatment plans.

### 4.2. Comparative analysis of existing biomarkers with SMAD4

The expression and mutational status of the SMAD4 protein has been widely discussed, with the hope that it could serve as a biomarker in the clinical research and practice of CRC, potentially aiding in the identification of patient subgroups at risk of early relapse after treatment.^68–70^ Recent studies have shown that the expression levels and mutations of SMAD4 are significantly associated with the prognosis of CRC patients, including OS and DFS.^2,34^ Related studies indicate that the mutation frequency of SMAD4 in CRC is approximately 10%, making it one of the most common mutated genes in CRC.^2^ Additionally, the loss of SMAD4 occurs at a rate of up to 30%.^32^ To fully understand its value in disease diagnosis, prognostic assessment, and treatment response, a comparative analysis with other typical biomarkers is beneficial (Table 1).

**Table 1.** Comparative analysis of key biomarkers in CRC.

Biomarker	Mutation/Expression Frequency (in CRC)	Role in CRC	Prognostic Value	Clinical Significance
SMAD4^64^	About 10%	Plays a regulatory role, related to tumor aggressiveness and metastatic potential	Indicates tumor aggressiveness, potential for survival prediction	Crucial for understanding the complex interactions within the tumor microenvironment
KRAS^81^	35−45%	Common mutation in CRC, critical for determining eligibility for EGFR-targeted therapies	Limited predictive value for survival; does not predict treatment response accurately	Essential for deciding eligibility for EGFR-targeted therapies
NRAS^72^	3−5% (Much lower than KRAS)	Similar role to KRAS in CRC	Similar to KRAS	Affects eligibility for EGFR-targeted treatments
BRAF^82^ (V600E）	8%−12% in metastatic CRC (mCRC)	Less common, associated with poor prognosis	Associated with poorer outcomes, does not respond well to anti-EGFR treatments	Forms the basis for developing targeted therapies
CA19-9^83^	Not applicable (serological marker)	Used for disease monitoring and assessing recurrence risk	Limited sensitivity and specificity, especially in early CRC diagnosis	Commonly used in monitoring, not suitable for early CRC screening

Compared to SMAD4, KRAS and NRAS gene mutations are among the most common genetic alterations in CRC, with KRAS mutations occurring at a frequency of approximately 35–45%^71^ and NRAS mutations occurring at a frequency of approximately 3–5%.^72^ These mutations are crucial in determining whether patients are suitable candidates for targeted EGFR therapy, and play a critical role in determining whether a patient is eligible for EGFR-targeted treatments. Although KRAS and NRAS mutations have certain advantages in screening and early diagnosis, their value as single prognostic indicators is limited because they cannot accurately predict survival rates or treatment response in all patients.^73,74^ In contrast, alterations in SMAD4 not only involve signal pathway regulation but are also closely related to tumor aggressiveness and metastatic potential, offering deeper insights into the biological behavior of the disease.

BRAF mutations, particularly V600E, are less common in CRC, accounting for approximately 8%-12%^75^ in metastatic CRC (mCRC), but typically indicate a poorer prognosis. BRAF mutations suggest that patients are unlikely to benefit from anti-EGFR treatments,^76^ and this mutation has become the basis for the development of new treatment strategies, such as targeted therapy for BRAF V600E.^77^ Unlike BRAF, alterations in SMAD4 more reflect the invasiveness and metastatic potential of the tumor, rather than being a direct predictor of response. Research on SMAD4 emphasizes its regulatory role in the development of CRC, particularly with respect to the tumor microenvironment and the interactions between tumor cells and their environment.^57^

CA19-9 is the most commonly used serologic marker in clinical practice for disease monitoring^78^ and assessment recurrence risk in CRC.^79^ However, the sensitivity and specificity of CA19–9 are limited, especially in early CRC diagnosis and screening. In contrast, changes in SMAD4 are directly related to the molecular and cellular mechanisms of the tumor, providing more in-depth information on disease pathology and having potential value in understanding tumor aggressiveness and predicting treatment responses.^80^

Overall, the uniqueness of SMAD4 compared to other biomarkers lies in its ability to reflect the molecular pathological features and biological behavior of CRC. Although it may not be as intuitive as some markers (such as CA19–9) in early diagnosis and screening, SMAD4 provides indispensable value in prognostic assessment and guiding the selection of personalized treatment plans.

## 5. SMAD4 protein as a therapeutic target for CRC and its targeted treatment strategies

As a key molecular marker in CRC, the critical role of SMAD4 in tumor initiation and development makes it a potential therapeutic target.

One potential treatment strategy to address SMAD4 dysfunction or mutation involves functional replacement or repair of SMAD4. Although there are currently no methods to directly restore SMAD4 function in clinical practice, research into gene therapy or small molecule drugs that activate the SMAD4 pathway is a promising direction.^84–86^ For example, the development of small molecule compounds that can stabilize SMAD4 protein expression, prevent its degradation, or enhance its transcriptional activity. Studies have indicated that circ_ITGA7 binds to miR-766, preventing miR-766 from degrading SMAD4 mRNA, thereby stabilizing and increasing SMAD4 expression to inhibit colorectal cancer (CRC) progression.^87^ Furthermore, research has discovered that the circular RNA hsa_circ_0004872 acts as a “molecular sponge” for miR-224, sequestering and inhibiting its activity. Since miR-224 suppresses SMAD4 expression, hsa_circ_0004872 indirectly promotes SMAD4 expression, thereby inhibiting the progression of gastric cancer, providing insights for CRC treatment by enhancing SMAD4 expression.^88^ Additionally, studies have shown that WIP1 dephosphorylates SMAD4 at the Thr277 site, reducing SMAD4 stability and accumulation in the nucleus, which in turn promotes cancer development. Thus, we hypothesize that promoting SMAD4 phosphorylation at the Thr277 site could inhibit CRC progression.^89^ In CRC, DUSP4 is highly expressed and promotes SMAD4 degradation through ubiquitination pathways. By regulating SMAD4 gene expression, DUSP4 enhances cell proliferation, migration, and invasion. This suggests that developing DUSP4 inhibitors could potentially reduce SMAD4 degradation, thereby inhibiting CRC progression.^35^

Given the central of SMAD4 role in TGF-β signaling, another strategy is to combat the malignant effects caused by SMAD4 abnormalities by inhibiting the entire pathway.^15^ Research has been conducted to develop inhibitors targeting TGF-β ligands, receptors, and downstream signaling molecules, thereby indirectly regulating SMAD4 function and inhibiting tumor cell proliferation, invasion, and immune evasion.^90,91^ Studies have shown that after the ingestion of black raspberries, gut microbiota convert them into bioactive benzoate metabolites. These metabolites can increase Smad4 expression in natural killer cells and colorectal cells through specific signaling pathways, thereby inhibiting colorectal cancer progression. These metabolites are also produced during the gut metabolism of many common plant-based foods, such as fruits, vegetables, and whole grains. Therefore, developing similar compounds could potentially inhibit colorectal cancer progression.^92^

xConsidering the high heterogeneity and complex microenvironment of CRC, targeting SMAD4 alone may not suffice for effective treatment. Therefore, combining SMAD4-targeted therapy with other treatment modalities such as chemotherapy, radiotherapy, immunotherapy, or drugs targeting other critical pathways (such as KRAS) could enhance the overall therapeutic effect.

## 6. Conclusion

In summary, although direct targeting of the SMAD4 protein is still in the exploratory stage, an increasing number of studies recognize the key role of SMAD4 and its signaling pathways in CRC. Future research should focus on developing and validating small molecule inhibitors that activate the SMAD4 pathway, as well as the clinical efficacy of combining these inhibitors with existing immune checkpoint inhibitors, providing new hope for personalized and precision treatment and prognosis of CRC patients. With continuous advancements in molecular biology and clinical trials, the effectiveness and safety of these strategies will be further validated and improved in the future.
